# Portable Deep Learning-Driven Ion-Sensitive Field-Effect Transistor Scheme for Measurement of Carbaryl Pesticide

**DOI:** 10.3390/s22093543

**Published:** 2022-05-06

**Authors:** Nongluck Houngkamhang, Pattarapong Phasukkit

**Affiliations:** 1 College of Materials Innovation and Technology, King Mongkut’s Institute of Technology Ladkrabang, Bangkok 10520, Thailand; nongluck.ho@kmitl.ac.th; 2 School of Engineering, King Mongkut’s Institute of Technology Ladkrabang, Bangkok 10520, Thailand

**Keywords:** deep learning, ISFET, carbaryl, acetylcholinesterase

## Abstract

This research proposes a multiple-input deep learning-driven ion-sensitive field-effect transistor (ISFET) scheme to predict the concentrations of carbaryl pesticide. In the study, the carbaryl concentrations are varied between 1 × 10^−7^–1 × 10^−3^ M, and the temperatures of solutions between 20–35 °C. To validate the multiple-input deep learning regression model, the proposed ISFET scheme is deployed onsite (a field test) to measure pesticide concentrations in the carbaryl-spiked vegetable extract. The advantage of this research lies in the use of a deep learning algorithm with an ISFET sensor to effectively predict the pesticide concentrations, in addition to improving the prediction accuracy. The results demonstrate the very high predictive ability of the proposed ISFET scheme, given an MSE, MAE, and R^2^ of 0.007%, 0.016%, and 0.992, respectively. The proposed multiple-input deep learning regression model with signal compensation is applicable to a wide range of solution temperatures which is convenient for onsite measurement. Essentially, the proposed multiple-input deep learning regression model could be adopted as an effective alternative to the conventional statistics-based regression to predict pesticide concentrations.

## 1. Introduction

Pesticides are substances that are commonly used in agriculture to deter, incapacitate, kill, or discourage pests while increasing crop yields. Along with these benefits, pesticides are toxic to humans and other species. Pesticides can translocate to cultivated crops and contaminate the environment. Furthermore, excessive use of pesticide and pesticide residues in fruit and vegetables prove harmful to human health [1,2].

Of particular concern is carbaryl, which is a broad-spectrum N-methyl carbamate insecticide widely used to control agricultural pests, such as aphids, fire ants, fleas, ticks, and spiders. Human exposure to carbaryl occurs through residues in food and skin contact [3,4]. Long-term carbaryl exposure can also cause blurred vision, vomiting, abdominal pain, breathing difficulty, and disruption of the nervous system [5,6,7].

Carbaryl is toxic to humans as the chemical inhibits acetylcholinesterase (AChE) enzyme activity, which disrupts the transmission of the nerve system. AChE is the cholinesterase enzyme primarily found in the neuromuscular junctions of insects and vertebrates, including humans [8,9,10,11]. AChE catalyzes the hydrolysis of acetylcholine (AChCl) to choline and acetic acid (CH_3_COOH). CH_3_COOH consists of four hydrogen atoms, but only one hydrogen bonded to oxygen is ionizable, yielding hydrogen ion (H^+^).

The advanced quantitative techniques commonly used to detect pesticide residues in fresh produce are high-performance liquid chromatography (HPLC) [12,13] and gas chromatography (GC) [14,15]. It should be underlined that the analytes can be detected in very low concentrations in these techniques. However, the advanced detection techniques encounter certain limitations, including costliness, high complexity, lengthy analysis, highly skilled personnel requirement, and unsuitability for onsite (field test) measurement.

To overcome the limitations inherent in the advanced detection techniques, the small size of the sensor is an advantage to its application for pesticide detection. The ISFET sensor is one of the alternative techniques utilized in this field due to its robustness and ease of integration, requiring little routine maintenance. Unlike other conventional sensors, ISFET probes can easily be inserted into samples such as fruit and vegetables. These are the reasons that ISFET is an appealing transduction platform. The ISFET sensor is a sensing device capable of measuring H^+^ in solution [16,17]. Due to its high sensitivity, rapid response, and compact size, the ISFET sensor is commonly used in biochemical analysis [18]. Additionally, the ISFET form factor is compact, energy-efficient, and affordable, rendering it highly suitable for onsite (field test) measurement [19,20,21,22]. However, the device suffers from inherent nonidealities (i.e., temperature drift), which considerably affect its performance due to the characteristic of the semiconductor substrate [23]. There have been works that use machine learning to compensate for sensor readout, in which the complex system or circuit design to reduce signal drift can be neglected [24,25,26,27]. The pesticide measurement using the ISFET sensor is carried out by immobilizing the AChE enzyme onto the ISFET sensing area before reacting with the AChCl substrate to obtain choline and CH_3_COOH. The ISFET sensor detects changes in H^+^ in the solution, which in turn induces changes in voltage between the gate and the source (i.e., ISFET signal response). Carbaryl inhibits the reaction between the AChE enzyme and the AChCl substrate, resulting in a decrease in H^+^ in the solution [11].

The ISFET sensor schemes with machine learning-based support vector machine (SVM) and artificial neural network (ANN) are used to predict pH values in solutions. The results show that the SVM model outperforms the ANN model. In Ref. [28], ANN is utilized to improve the prediction accuracy of the ISFET sensor. Furthermore, the application of machine learning to biosensors to enhance the performance of sensors is on a continual rise. Particularly, machine learning has been used for imaging, electronic-nose and electronic-tongue, and surface-enhanced Raman spectroscopy (SERS) biosensors [29,30,31].

In Ref. [32], InGaZnO-based Electrolyte-Gate Field-Effect Transistors (EGFET) is used for glucose sensor by applying machine learning-based techniques to enhance the accuracy of data detection. Although EGFET is small and highly sensitive, a custom material design and optimization are required [33]. Other papers have reported using an ISFET sensor for environmental monitoring [34,35]. Still, a published article has not written the application of deep learning in measuring pesticides based on enzyme inhibition assay using an ISFET sensor. Typically, an enzyme-ISFET sensor is used for pesticide measurements at room temperature. Due to the robustness, compact size, low power consumption, and high sensitivity of ISFET, it is an alternative technology for development as the screening device of samples for pesticide in field testing. The temperature can affect the enzyme activity; enzymes work differently at different temperatures that should not exceed 40 °C. Temperatures also affect the ISFET sensor, which can cause signal drift and may affect the accuracy of pesticide concentrations. Therefore, this work is interested in developing a technology platform of ISFET combined with a deep learning model for measuring pesticide concentration at any temperature in 20–35 °C. The developed deep learning regression model can improve the prediction accuracy of pesticide concentration and could be easily deployed on onsite screening devices due to the feed-forward computing in deep learning processes. Pesticide concentrations can be measured at any temperature (20–35 °C) without complicated programming.

The commercialized ISFET is used in this research to obtain a relatively stable and reliable device. The platform technology (ISFET combined with the deep learning model) presented here is more flexible than the conventional testing in the laboratory. It has the potential to be the quick and inexpensive screening of samples for pesticides. This will strengthen the utilization of ISFET sensors in pesticide detection, improve consumer demand for safe foods, and increase the trading value of vegetables and fruits tested to be organic products.

To demonstrate the detection of carbaryl pesticide by ISFET sensor at various temperatures in the range of 20–35 °C, which resemble the ambient temperatures in tropical countries, the machine learning is utilized to simplify signal compensation and improve the accuracy of predicted carbaryl concentration. Due to the variation of enzyme immobilization on different ISFET sensors, the uncertainty yields different sensor values. The variation of ISFET reference signals have been compensated through machine learning techniques.

Specifically, this research proposes an ISFET scheme driven by multiple-input deep learning regression with signal compensation to predict carbaryl concentrations at different temperatures. Based on the ISFET signal response and the solution temperature, the prediction performance of the proposed multiple-input deep learning regression is reported in terms of mean squared error (MSE), mean absolute error (MAE), and correlation (R^2^). In addition, the proposed multiple-input deep learning regression is deployed to a portable device for onsite application by measuring the spiked carbaryl concentrations in ethanolic vegetable extract solution and comparing the results with the actual concentrations.

## 2. Materials and Research Methodology

### 2.1. Materials and Methods

#### 2.1.1. ISFET Sensor

In this research, the ISFET sensor was acquired from the Thai Microelectronics Center (TMEC), National Electronics and Computer Technology Center (NECTEC), Thailand. The ISFET sensor is of complementary metal-oxide-semiconductor (CMOS) technology, with silicon nitride (Si_3_N_4_) as the gate electrode, with the width (W) and the length (L) of the sensing area of 2.0 and 0.1 mm, respectively. The sensing area measures H^+^ in the solution as the gate material (Si_3_N_4_) donates and accepts protons.

Prior to the measurement, the ISFET sensor is connected to the silver/silver chloride (Ag/AgCl) reference electrode to measure the ISFET signal response in the solution. The ISFET signal response is the potential difference at the interface between the gate electrode and solution, which varies in response to H^+^ in the solution [36,37].

#### 2.1.2. Preparation of Experimental Solutions and Pesticide

The experimental solutions are phosphate-buffered saline (PBS) and acetylcholine chloride (AChCl) substrate, and the pesticide is carbaryl of varying concentrations. In the PBS preparation, 4.0 g of sodium chloride (NaCl), 1.45 g of disodium hydrogen phosphate (Na_2_HPO_4_), 0.1 g of potassium dihydrogen phosphate (KH_2_PO_4_), and 0.1 g of potassium chloride (KCl) are dissolved in 500 mL of deionized (DI) water for 5 mM PBS buffer with pH of 7.4. The pH of the PBS buffer is maintained at 7.4 using sodium hydroxide.

In the AChCl substrate preparation, AChCl (Sigma-Aldrich, St. Louis, MI, USA) is dissolved in PBS buffer for 5 mM AChCl substrate to react with the acetylcholinesterase (AChE) enzyme. In the carbaryl pesticide preparation, carbaryl (in powder form; Sigma-Aldrich, St. Louis, MI, USA) is first dissolved in 100% ethanol for the stock solution of carbaryl before diluting in PBS solution containing 5% ethanol. The experimental concentrations of carbaryl are 1 × 10^−7^, 1 × 10^−6^, 1 × 10^−5^, 1 × 10^−4^, and 1 × 10^−3^ M.

#### 2.1.3. pH Sensitivity Test

Prior to the measurement of carbaryl, the pH sensitivity of the ISFET sensor is calibrated by testing in pH buffer (Merck, Darmstadt, Germany) of different pH values (pH 4, 7, and 10), where pH 4, 7, and 10 represent acidity, neutrality, and alkalinity. In the sensitivity test, the ISFET sensor with Ag/AgCl reference electrode is sequentially immersed into three beakers containing the pH buffer of different pH values (pH 4, 7, and 10 for the first, second, and third beakers, respectively). Specifically, in one cycle of sensitivity test, the ISFET sensor is immersed in the first beaker (pH 4) for one minute before transferring to the second beaker (pH 7) for one minute and then to the third beaker (pH 10) for another minute; the cycle is conducted three times. The ISFET signal responses under different pH values are collected. The sensitivity of the ISFET sensor is the slope of the plot of the ISFET signal response relative to pH value, given the acceptable sensitivity of 45–55 mV/pH.

#### 2.1.4. Measurement of Carbaryl Concentrations

**Figure 1:**
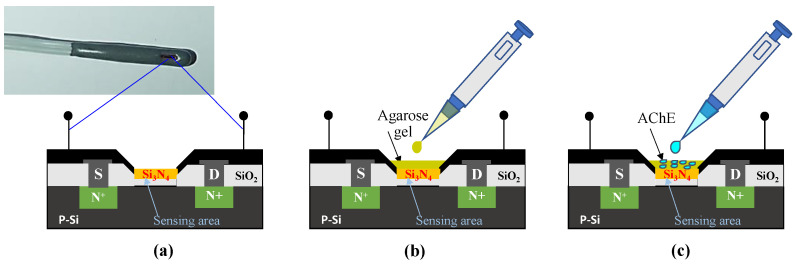
Schematic of the ISFET surface preparation: (**a**) ISFET sensing area, (**b**) application of agarose gel, and (**c**) enzyme immobilization.

Prior to the measurement, the ISFET sensing area is cleaned with isopropanol and DI water and left to dry. Afterward, 2.0 µL agarose gel (2.0% *w/v* in DI water) is applied to the sensing area and left for 15 min to dry before dropping (i.e., immobilizing) 1 unit of AChE enzyme as shown in Figure 1a–c. The ISFET sensor containing the AChE enzyme is then left to dry at room temperature before refrigerating at 4 °C for 24 h.

After 24 h, the AChE-coated ISFET sensor is immersed in beakers containing PBS buffer for 3 min (180 s), given that the ISFET signal response remains unchanged after 3 min. The temperatures of the PBS buffer are varied between 20, 25, 30, and 35 °C (T1–T4, respectively). The baseline ISFET signal response data (i.e., baseline signal response) under various PBS temperatures are collected. Afterward, the AChE-coated ISFET sensor is transferred to beakers containing AChCl substrate for another 3 min (180 s) [38], with the corresponding AChCl substrate temperatures of 20, 25, 30, and 35 °C (T1–T4, respectively). The ISFET signal response under various AChCl substrate temperatures is then collected.

In this study, ΔV_gs_^w/o carbaryl^ is the ISFET signal response without carbaryl (i.e., reference signal response), which is the difference between the baseline signal response and the signal response in AChCl substrate, given T1–T4. The experiments are carried out in triplicate.

Once the ΔV_gs_^w/o carbaryl^ data collection is complete, 5 µL of carbaryl diluted with PBS buffer containing 5% ethanol is pipetted onto the sensing area of the AChE-coated ISFET sensor and incubated for 5 min. Likewise, 5 µL of carbaryl diluted with ethanolic vegetable extract is applied onto the sensing area of the AChE-coated ISFET sensor and incubated for 5 min. In this study, the PBS-diluted and vegetable extract-diluted carbaryl concentrations are 1 × 10^−7^, 1 × 10^−6^, 1 × 10^−5^, 1 × 10^−4^, and 1 × 10^−3^ M. The experimental vegetable is organically grown white cabbage (*Brassica oleraceae var. capitata f. alba*). In the vegetable extraction, white cabbage is cut into small shreds of 0.5 × 0.5 cm (W × L) in dimension, extracted by ethanol, and filtered by Whatman No. 1 for vegetable extract. According to [39], carbaryl is commonly used to deter cabbage worms (*Pieris rapae*) in white cabbage due to its high efficacy.

For both the PBS-diluted and vegetable extract-diluted carbaryl experiments, the carbaryl-incubated ISFET sensor is then immersed in beakers containing PBS buffer for 3 min (180 s), given the PBS temperatures of 20, 25, 30, and 35 °C (T1–T4, respectively). The carbaryl-incubated baseline ISFET signal response data under various PBS temperatures are collected. Afterward, the carbaryl-incubated ISFET sensor is transferred to beakers containing AChCl substrate for another 3 min (180 s), with the corresponding AChCl substrate temperatures of 20, 25, 30, and 35 °C (T1–T4, respectively). The ISFET signal response under various AChCl substrate temperatures is also collected. The ISFET signal response with carbaryl (ΔV_gs_^carbaryl^) is the difference between the carbaryl-incubated baseline signal response and the signal response in the AChCl substrate, given T1–T4.

### 2.2. Deep Learning Regression-Based Models with Signal Compensation

**Figure 2:**
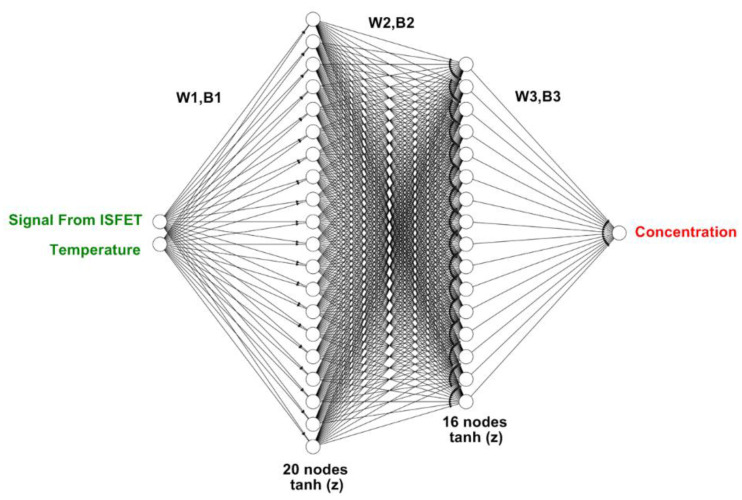
The multiple-input deep learning regression-based model predicts pesticide concentration based on the ISFET signal response and solution temperature.

Figure 2 depicts the multiple-input deep learning regression-based model to predict the pesticide (i.e., carbaryl) concentration based on the ISFET signal response and the solution temperature. The model inputs (features) include the signal response from the ISFET sensor and the temperatures of solutions (i.e., PBS buffer and AChCl substrate). The model output (target) is the concentrations of carbaryl. The algorithmic model consists of two hidden layers, with 20 and 16 nodes in the first and second hidden layers, respectively. The numbers of nodes in both hidden layers are doubled, vis à vis the single-input deep learning model, to enhance the predictive ability of the multi-input model. The W and B between layers (W1, B1, W2, B2, W3, and B3) are optimized by gradient descent iterative optimization algorithm. The activation function of the two hidden layers is hyperbolic tangent function (tanh(z)), and that of the output layer is rectified linear unit (ReLU) activation function.

In this research, the carbaryl concentrations are varied between 1 × 10^−7^ and 1 × 10^−3^ M (i.e., 1 × 10^−7^, 1 × 10^−6^, 1 × 10^−5^, 1 × 10^−4^, and 1 × 10^−3^ M) and the temperatures of solutions between 20, 25, 30, and 35 °C (T1–T4, respectively). The experimental solutions are PBS buffer and AChCl substrate. The experiments are carried out 10 times for each temperature. The initial number of datasets for training and testing the multiple-input algorithmic model is thus 200 datasets (5 carbaryl concentrations × 4 solution temperatures × 10 times). To improve the predictive ability of the model, the number of datasets is mimicked 10 times using additive white gaussian noise (AWGN) to 2000 datasets.

This is to increase the number of data sets by mimicking the noise with AWGN to relate to the noise that can naturally occur from the measurement, such as thermal noise, electronic noise, sunlight, etc. [40]. In deep learning development, AWGN are added to the signal measurement of training data. For AI to learn the signals that correspond to actual use, AI can still be effective in work. Refs. [41,42] support that increasing datasets by AWGN can eliminate noisy versions of the signal to train and test the model.

The additive datasets (2000 datasets) are subsequently divided into two groupings: training (80%) and testing groupings (20%). As a result, the training and testing groupings consist of 1600 and 400 datasets. Specifically, for each solution temperature (T1–T4), there are 400 training datasets, giving rise to a total of 1600 training datasets (400 × 4 solution temperatures). Likewise, there are 100 testing datasets for each solution temperature.

The training dataset comprises *X_train_* (normalized training input dataset) and *Y_train_* (normalized training output dataset). The testing dataset consists of *X_test_* (normalized testing input dataset) and *Y_test_* (normalized testing output dataset). The initial randomized W1, B1, W2, B2, W3, and B3 are optimized by a gradient descent iterative optimization algorithm with the given learning rate (α) and epoch of 0.01 and 5000. The iteration is terminated once divergence occurs between the MSE of the training and testing datasets.

Prior to training and testing the multiple-input deep learning models, the ISFET signal response without carbaryl (ΔV_gs_^w/o carbaryl^) or reference signal is needed to compensate for solving the variation of different AChE-coated ISFET sensors [43,44,45]. In addition, the voltage for the calibrated sensor is required to determine the reference signal compensation. To compensate the reference signal is the first step before implementing the signal into the multiple-input deep learning regression model.

**Figure 3:**
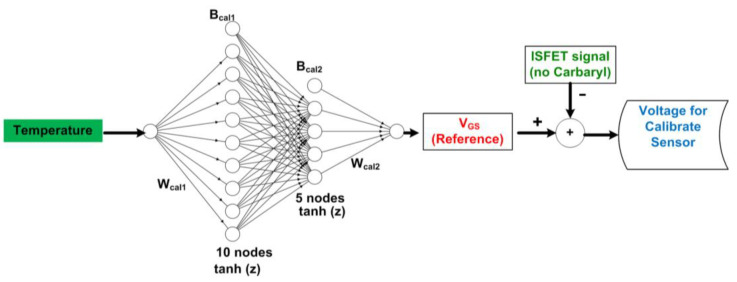
The single-input deep learning regression-based model calculates the voltage for calibrating the sensor.

To determine the voltage for the calibrated sensor, the data of reference signals (ΔV_gs_^w/o carbaryl^) have been collected at various temperatures (20, 25, 30, and 35 °C). The single-input deep learning model of the relation between temperature and reference signals is created (Figure 3).

When applying the AChE-coated ISFET sensors to detect carbaryl, the ISFET signal (no carbaryl) is needed to be measured. We apply the signal to the model in which the temperature of that signal and the V_gs_ (reference) will be obtained. Then, the ISFET signal (no carbaryl) is compared to the obtained V_gs_ (reference) to calculate the voltage for calibrated sensor and then implemented to the multiple-input deep learning regression model.

The single-input deep learning model to calculate the voltage for a calibrated sensor for signal compensation is shown in Figure 3. The algorithmic model consists of two hidden layers, with 10 and 5 nodes in the first and second hidden layers, respectively. The initial randomized weight (W) and bias (B) between layers (i.e., W_cal1_, B_cal1_, W_cal2_, and B_cal2_) are optimized by gradient descent iterative optimization algorithm, adjusted weight, and bias with gradient descent number of learning rate 0.01 and epoch 3000. The iteration is terminated once divergence occurs between the MSE of the training and testing datasets or the MSE equal to 0.01. The number of datasets equals the multiple-input deep learning regression model (2000 datasets).

Prior to training and testing the deep learning models, the training and testing input and output datasets (*X_train_*, *Y_train_*, *X_test_*, and *Y_test_*) under variable concentrations of carbaryl (1 × 10^−7^, 1 × 10^−6^, 1 × 10^−5^, 1 × 10^−4^, and 1 × 10^−3^ M), given a specific solution temperature (T1–T4), are normalized using min–max normalization in Equation (1).

(1) $$Data_{normalization}=\frac{\left(Dataset-Dataset_{min}\right)}{\left(Dataset_{max}-Dataset_{min}\right)}$$

where *Dataset* is the training and testing input and output datasets (i.e., *X_train_*, *Y_train_*, *X_test_*, and *Y_test_*); *Dataset_min_* is the minimum dataset of the training input and output datasets (*X_train_* and *Y_train_*), and the minimum dataset of the testing input and output datasets (*X_test_* and *Y_test_*); and *Dataset_max_* is the maximum dataset of the training input and output datasets (*X_train_* and *Y_train_*) and the maximum dataset of the testing input and output datasets (*X_test_* and *Y_test_*). The value of the normalized datasets (*Data_normalization_*) is between 0 and 1 [0, 1].

In the feedforward of the deep learning models, the activation function between hidden layers is a hyperbolic tangent function (tanh(z)), as shown in Equation (2), where tanh(z) = [−1, 1]. The activation function ReLU(z) is used in the output layer, as shown in Equation (3), where z is the linear combination (Equation (4)). The predicted output ${\hat{y}}_{n}$ of the single-input deep learning regression-based model is the V_gs_ (reference), and that (predicted) of the multiple-input deep learning model is the carbaryl concentration.

(2) $$\tanh(z)=\frac{\left(e^{z}-e^{-z}\right)}{\left(e^{z}+e^{-z}\right)}$$

(3) $$\mathrm{ReLU}\left(z\right)=\{\begin{array}{c} 0,\;z<0\\ z,\;z\geq 0 \end{array}$$

(4) $$Z=\left[\begin{array}{c} z_{1}\\ z_{2}\\ \begin{array}{c} \vdots \\ z_{N} \end{array} \end{array}\right]=\left[\begin{array}{ccc} \begin{array}{c} x_{1}^{1}w_{1}\\ x_{2}^{1}w_{1}\\ \begin{array}{c} \vdots \\ x_{N}^{1}w_{1} \end{array} \end{array} & \begin{array}{c} x_{1}^{2}w_{2}\\ x_{2}^{2}w_{2}\\ \begin{array}{c} \vdots \\ x_{N}^{2}w_{2} \end{array} \end{array} & \begin{array}{cc} \begin{array}{c} \ldots \\ \ldots \\ \begin{array}{c} \vdots \\ \ldots \end{array} \end{array} & \begin{array}{c} x_{1}^{D}w_{D}\\ x_{2}^{D}w_{D}\\ \begin{array}{c} \vdots \\ x_{N}^{D}w_{D} \end{array} \end{array} \end{array} \end{array}\right]+\left[\begin{array}{ccc} B_{1} & B_{2} & \begin{array}{cc} \ldots & B_{D} \end{array} \end{array}\right]$$

In the back propagation of deep learning models, the mean squared error (MSE) between the normalized training output dataset (*Y_train_*; y_n_) and predicted normalized output ${\hat{y}}_{n}$ is first calculated using Equation (5), and the gradient descent iterative optimization algorithm is subsequently applied to fine-tune W and B by using Equation (6) and the chain rule derivative.

(5) $$\mathrm{MSE}=\frac{1}{n}\;\sum_{i\;=1}^{n}{\left(y_{n}-{\;\hat{y}}_{n}\right)}^{2}$$

(6) $$\frac{\partial \mathrm{MSE}}{\partial W_{i}}\;\mathrm{and}\;\frac{\partial \mathrm{MSE}}{\partial B_{i}}$$

where *i* = 1, 2, 3 corresponding to W1, B1, W2, B2, W3, and B3, respectively, and the derivative of tanh(z) activation function for hidden layers is expressed in Equation (7).

(7) $$\frac{\partial \left[\tanh(\mathrm{Az})\right]}{\partial z}=1-\tanh z^{2}$$

The prediction performance (i.e., predictive ability) of the multiple-input deep learning regression-based model is assessed by mean squared error (MSE; Equation (5)), mean absolute error (MAE; Equation (8)), and coefficient of determination (R^2^; Equation (9)).

(8) $$\mathrm{MAE}=\frac{1}{n}\;\sum_{i\;=1}^{n}\left|y_{n}-{\;\hat{y}}_{n}\right|$$

where y_n_ is the normalized testing output dataset (*Y_test_*), ${\hat{y}}_{n}$ is predicted normalized output (*Y_predict_*), and n is the number of datasets.

The coefficient of determination (R^2^) is a goodness-of-fit measure for regression models and the dependent variables on a scale of 0–1.

(9) $$R^{2}=\frac{\left(\mathrm{Var}\left(Y\right)-\mathrm{MSE}\right)}{\mathrm{Var}\left(Y\right)}$$

where Var is the mean of differences between y_n_ and average (y_n_) squared and MSE is expressed in Equation (5).

**Figure 4:**
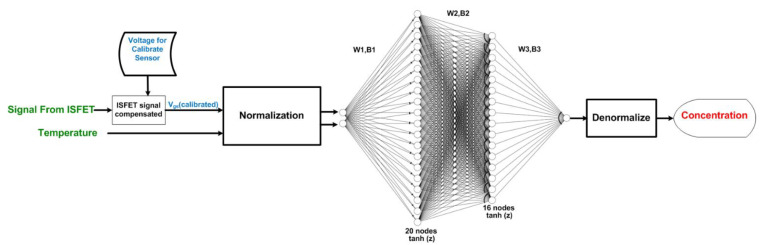
The process of signal implementation on the multiple-input deep learning regression-based model for predicting the pesticide concentration.

Figure 4 shows the multiple-input deep learning regression model to predict the concentrations of carbaryl (target) based on the ISFET signal response and solution temperatures (features). In the proposed multiple-input deep learning model, the features are normalized prior to feedforwarding for the normalized target, given the optimized W1, B1, W2, B2, W3, and B3. The normalized target is subsequently denormalized to obtain the carbaryl concentration.

## 3. Experimental Setup and Data Analysis

### 3.1. Experimental Equipment Setup and Data Collection

In this research, a database of the datasets for training and testing the deep learning regression-based models is first created in the laboratory. The database consists of the ISFET signal response and the temperatures of solutions (PBS and AChCl substrate), given the concentrations of carbaryl of 1 × 10^−7^, 1 × 10^−6^, 1 × 10^−5^, 1 × 10^−4^, and 1 × 10^−3^ M.

**Figure 5:**
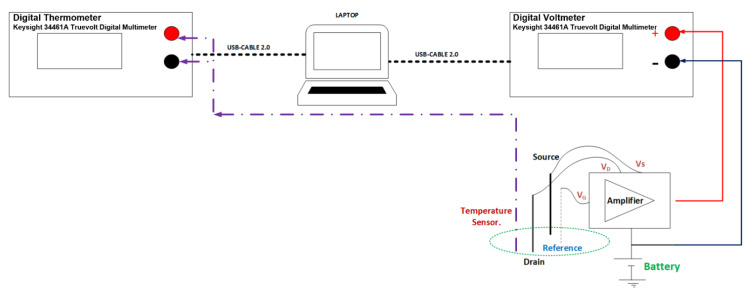
Diagram of the experiment setup and data collection, where V_G_, V_D_, and V_S_ denote gate voltage, drain voltage, and source voltage, respectively.

In Figure 5, the ISFET signal response data at various carbaryl concentrations (1 × 10^−7^–1 × 10^−3^ M) are collected using Keysight 34461A Truevolt Digital Multimeter. The ISFET signal response is amplified using Winsense (Winsense Co., Ltd., Bangkok, Thailand) and stored on a laptop computer in spreadsheet format. Meanwhile, the solution temperatures at different carbaryl concentrations are collected by Keysight 34461A Truevolt Digital Multimeter with a two-wire 10 kΩ thermistor temperature sensor and retained on the laptop computer in spreadsheet format. The data on the ISFET signal response and solution temperatures are collected in a concurrent fashion at one-second interval. Table 1 tabulates the specifications of the equipment and the ISFET sensor used in this research.

### 3.2. Calculation of Enzyme Inhibition by Carbaryl

The AChE enzyme inhibition by carbaryl of various concentrations (1 × 10^−7^–1 × 10^−3^ M), given the solution temperatures of 20, 25, 30, and 35 °C (T1–T4, respectively), is calculated by Equation (10).

(10) $$\mathrm{percentage}\;\mathrm{of}\;\mathrm{enzyme}\;\mathrm{inhibition}=\left|\frac{{\Delta V}_{\mathrm{gs}}^{w/o\;\mathrm{carbaryl}}-{\Delta V}_{\mathrm{gs}}^{\mathrm{carbaryl}}}{{\Delta V}_{\mathrm{gs}}^{w/o\;\mathrm{carbaryl}}}\right|\times 100$$

where ΔV_gs_^w/o carbaryl^ is the ISFET signal response without carbaryl (i.e., reference signal response), which is the difference between the baseline signal response and the signal response in AChCl substrate; and ΔV_gs_^carbaryl^ is the ISFET signal response with carbaryl, which is the difference between the carbaryl-incubated baseline signal response and the signal response in AChCl substrate.

### 3.3. Preparation of Training and Testing Datasets

The initial number of datasets for training and testing the deep learning algorithmic models is 200 datasets (5 carbaryl concentrations × 4 solution temperatures × 10 times). The carbaryl concentrations are varied between 1 × 10^−7^, 1 × 10^−6^, 1 × 10^−5^, 1 × 10^−4^, and 1 × 10^−3^ M and the temperatures of solutions between 20, 25, 30, and 35 °C (T1–T4, respectively). In this research, the solutions are PBS buffer and AChCl substrate. To improve the predictive ability of the models, the number of datasets is mimicked 10 times using AWGN to 2000 datasets.

The datasets for the deep learning model are divided into two groupings: training (80%) and testing (20%). The training and testing groupings thus consist of 1600 and 400 datasets. Specifically, for each solution temperature (T1–T4), there are 400 training datasets, resulting in a total of 1600 training datasets (400 × 4 solution temperatures). Additionally, there are 100 testing datasets for each solution temperature.

**Figure 6:**
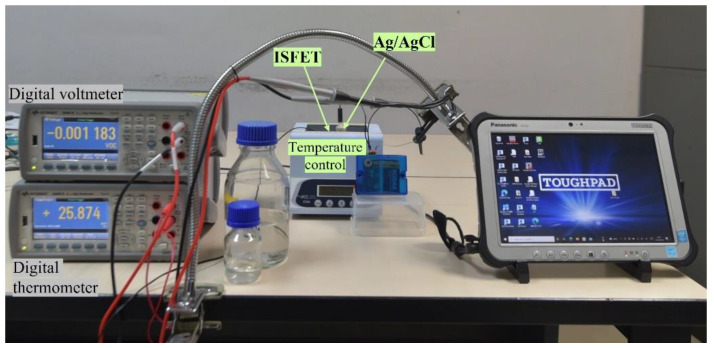
Laboratory experimental setup to collect the ISFET signal and temperature data.

Figure 6 shows the real-time data collection of ISFET signal response and solution temperature using the ISFET sensor and experimental equipment.

### 3.4. Onsite Application of the Multiple-Input Deep Learning Model

**Figure 7:**
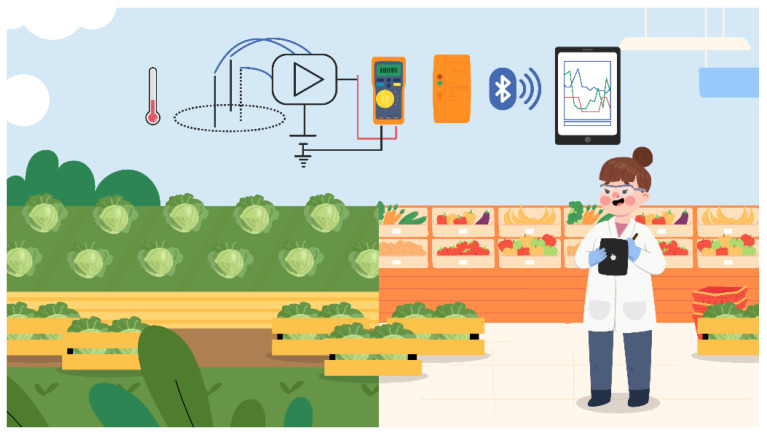
The experimental setup and onsite application of the multiple-input deep learning model.

Figure 7 illustrates the experimental setup and onsite application of the proposed multiple-input deep learning regression model using a portable digital multimeter (KEYSIGHT U1231A) outfitted with a real-time infrared (IR)-to-Bluetooth (BT) adapter (KEYSIGHT U1117A). The ISFET with reference electrode is connected to the portable digital multimeter with an IR-to-BT adapter. The optimized train parameters (W1, B1, W2, B2, W3, and B3) can be loaded onto the mobile device to construct a portable system. The compensated ISFET signal and temperature are normalized and fed to the deep learning algorithm model at the initial step. The output was calculated by the values of weight and bias applied in the feed-forward process. The result is a denormalized carbaryl concentration. From this context, the ISFET signal and temperature are sent to mobile devices that program the multiple-input deep learning regression model. This renders the onsite scheme economical and convenient to use.

In the actual application, the concentrations of carbaryl in fresh produce (i.e., white cabbage) are predicted based on the ISFET signal response and solution temperatures using the multiple-input deep learning regression model (Figure 4). In this research, the proposed multiple-input model is applied to determine the carbaryl concentrations in the ethanolic extract of organically grown white cabbage deliberately spiked with variable concentrations of carbaryl (1 × 10^−7^ to 1 × 10^−3^ M).

In the onsite implementation, the prediction performance of the proposed multiple-input ISFET scheme is validated by testing with the ethanolic extract of white cabbage spiked with various concentrations of carbaryl. The ex-ante (input) and ex-post (output) concentrations of carbaryl should closely resemble one another. The input refers to the carbaryl concentrations in the spiked vegetable extract and the output to the carbaryl concentrations displayed on a Bluetooth-enabled mobile device.

## 4. Results and Discussion

### 4.1. ISFET pH Sensitivity Result

**Figure 8:**
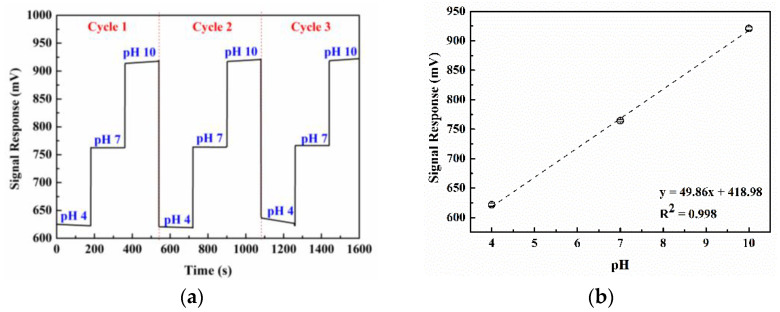
The pH sensitivity of ISFET sensor: (**a**) ISFET signal response given pH of 4, 7, and 10, (**b**) signal response relative to pH (mV).

Figure 8a illustrates the pH sensitivity of the experimental ISFET sensor in terms of signal response (mV) relative to time (s) at pH of 4, 7, and 10. (Please refer to Section 2.1.3 for the sequential procedure of the pH sensitivity test.) Figure 8b shows the linear relationship between the ISFET signal response (y) relative to the pH value (x), where y = 49.86x + 418.98, with R^2^ = 0.998. The slope of the linear relationship (i.e., the ISFET sensitivity) is 49.86 mV/pH. Given the acceptable ISFET sensitivity of 45–55 mV/pH, the experimental ISFET sensor can be deployed to measure the carbaryl concentrations.

### 4.2. Measurement of Concentrations of Carbaryl Diluted with PBS Buffer

**Figure 9:**
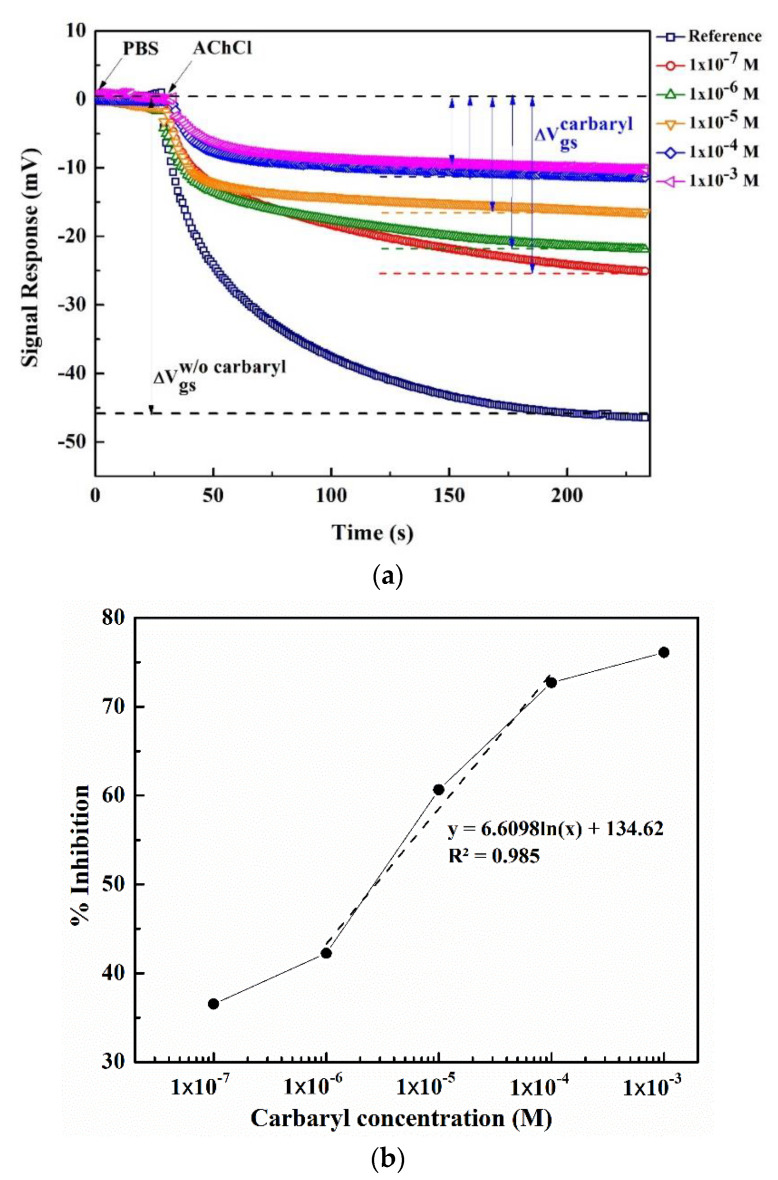
Carbaryl-induced AChE enzyme inhibition under variable carbaryl concentrations given 25 °C: (**a**) ISFET signal response without (ΔV_gs_^w/o carbaryl^; reference) and with carbaryl (ΔV_gs_^carbaryl^), (**b**) a corresponding percentage of AChE enzyme inhibition.

Figure 9a shows the ISFET signal response without carbaryl (ΔV_gs_^w/o carbaryl^) and with carbaryl (ΔV_gs_ ^carbaryl^) under variable carbaryl concentrations (1 × 10^−7^–1 × 10^−3^ M), given the solution temperature of 25 °C. (Please refer to Section 2.1.4 for the ISFET signal response measurement procedure). The experiments are carried out in triplicate, and the experimental solutions include PBS buffer and AChCl substrate.

Figure 9b illustrates the corresponding percentage of AChE enzyme inhibition under different carbaryl concentrations, given the solution temperature of 25 °C (i.e., standard reference ambient temperature). In order to achieve uniformity in measurements, 25 °C has been internationally adopted as the standard reference ambient temperature. This ISFET sensor can be detected carbaryl in the range of 1 × 10^−6^ to 1 × 10^−4^ M with a linear equation as y = 6.6098ln(x) + 134.62 and R^2^ = 0.985.

In Figure 9a, the mean and standard deviation (SD) of the ISFET signal response without carbaryl (ΔV_gs_^w/o carbaryl^) are 45.35 mV and 1.6 mV, respectively. Meanwhile, the measured ISFET signal response with carbaryl at the lowest experimental carbaryl concentration of 1 × 10^−7^ M ($\Delta V_{{\mathrm{gs}}_{minimum}}^{\mathrm{carbaryl}}$) is 28.78 mV. The limit of detection (LOD) of the ISFET sensor with the multiple-input deep learning regression is determined by Equation (11), the lowest experimental carbaryl concentration of 1 × 10^−7^ M ($\Delta V_{{\mathrm{gs}}_{minimum}}^{\mathrm{carbaryl}}$) (i.e., 28.78 mV) taking into account three standard deviations of the mean (3 × SD = 4.8 mV) is less than the ISFET signal response without carbaryl (ΔV_gs_^w/o carbaryl^) (i.e., <45.35 mV). The ISFET signal response of LOD calculated using Equation (11) is 33.50 mV. Given that 33.50 < 45.35 mV, the proposed scheme (i.e., ISFET sensor with the multiple-input deep learning regression) can measure the carbaryl concentrations in solutions as low as 1 × 10^−7^ M. Specifically, the LOD of the proposed ISFET scheme is 1 × 10^−7^ M (0.02 ppm) which is below the maximum residual limits (MRLs) of carbaryl in cabbage (0.05 ppm).

(11) $$\mathrm{LOD}\;((3\;\times \;\mathrm{SD})\;+\;\Delta V_{{\mathrm{gs}}_{minimum}}^{\mathrm{carbaryl}})<{\Delta V}_{\mathrm{gs}}^{w/o\;\mathrm{carbaryl}}$$

**Figure 10:**
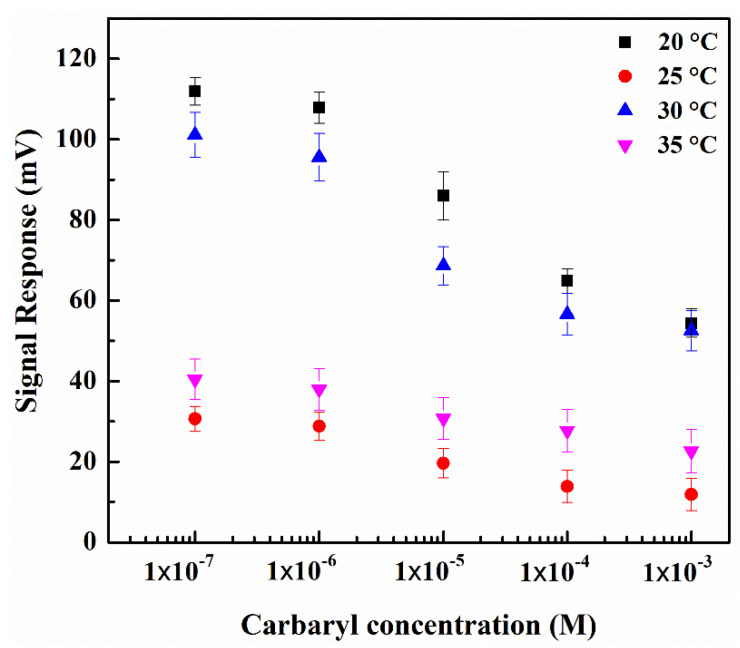
The ISFET signal response under variable concentrations of PBS-diluted carbaryl, given the solution temperatures of 20, 25, 30, and 35 °C.

Figure 10 illustrates the ISFET signal response relative to carbaryl concentrations under variable solution temperatures: 20, 25, 30, and 35 °C. The experimental solutions are PBS buffer and AChCl substrate. Essentially, the solution temperatures affect the carbaryl-induced AChE enzyme inhibition [46,47] and the subsequent ISFET signal response.

In addition, the effects of interferent compounds such as imidacloprid and chlorpyrifos were tested on the AChE-coated ISFET sensors (data not shown). The test showed no noticeable changes in the ISFET signal response detected in the presence of imidacloprid. Still, the observed ISFET signal response is changed in the presence of chlorpyrifos. The results indicate that chlorpyrifos can interfere with the determination of carbaryl because chlorpyrifos is the organo-phosphate pesticide that can inhibit the activity of AChE, unlike imidacloprid, an insecticide in a class of chemicals called the neonicotinoids that cannot inhibit the function of AChE with their substrate. Moreover, when applied to an actual sample test, the ionic strength, pH value, and interaction of some components of the analyzed sample on the enzyme activity may affect the ISFET signal.

### 4.3. Comparison of the Actual and Predicted Carbaryl Concentrations in PBS Buffer

**Figure 11:**
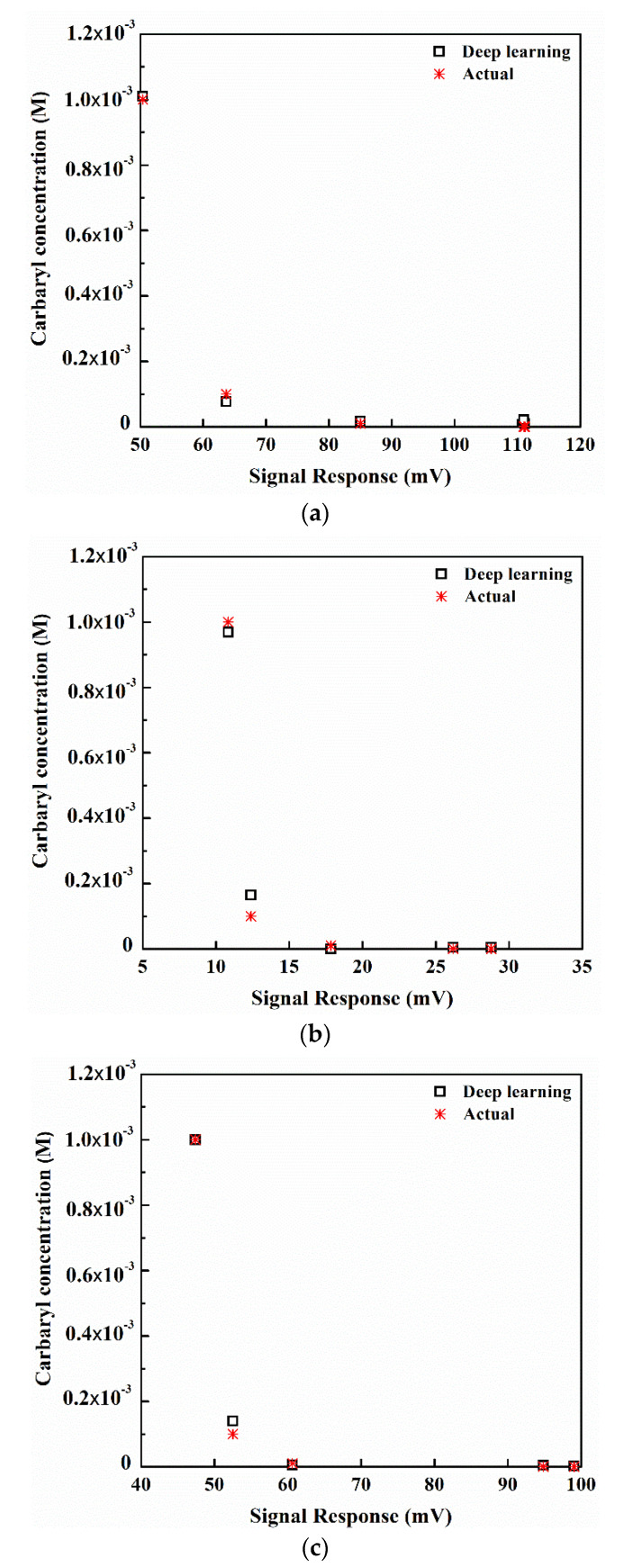

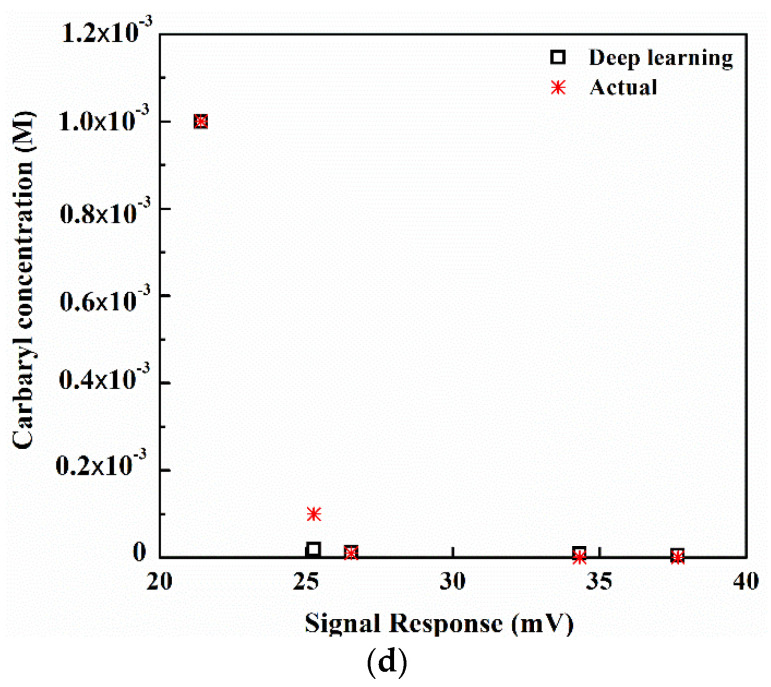
The actual and predicted concentrations of carbaryl in PBS using the multiple-input deep learning model at different temperatures of solutions given R^2^ = 0.992: (**a**) 20 °C, (**b**) 25 °C, (**c**) 30 °C, and (**d**) 35 °C.

Figure 11a–d compare the actual and predicted carbaryl concentrations using the multiple-input deep learning regression model, given the solution temperatures of 20, 25, 30, and 35 °C, respectively.

The MSE, MAE and R^2^ (Equations (5), (8), and (9), respectively) of the proposed multiple-input deep learning regression model are 0.007%, 0.016% and 0.992, respectively. Given the substantially low MSE and MAE (i.e., <1.0%), the multiple-input deep learning model possesses very high predictive ability and thus could be adopted to predict the pesticide concentrations in solutions. In addition, R^2^ of 0.992 shows a goodness-of-fit measure of the multiple-input deep learning regression model.

More importantly, the proposed multiple-input deep learning regression model is applicable to a wide range of solution temperatures in fractional (e.g., 20.5, 26.7, and 32.6 °C) and non-fractional increments (e.g., 20, 26, and 32 °C) which can deploy onsite testing.

### 4.4. Prediction of Vegetable Extract-Diluted Carbaryl Concentrations Using a Multiple-Input Deep Learning Model

In this section, the proposed multiple-input deep learning model is applied to predict the concentrations of carbaryl diluted with ethanolic extract of organically grown white cabbage. In this research, the multiple-input deep learning model is initially constructed using the datasets of the carbaryl concentrations diluted with PBS buffer. Therefore, the direct application of the initial multiple-input deep learning model (i.e., without input compensation) renders the prediction outcomes susceptible to error.

**Figure 12:**
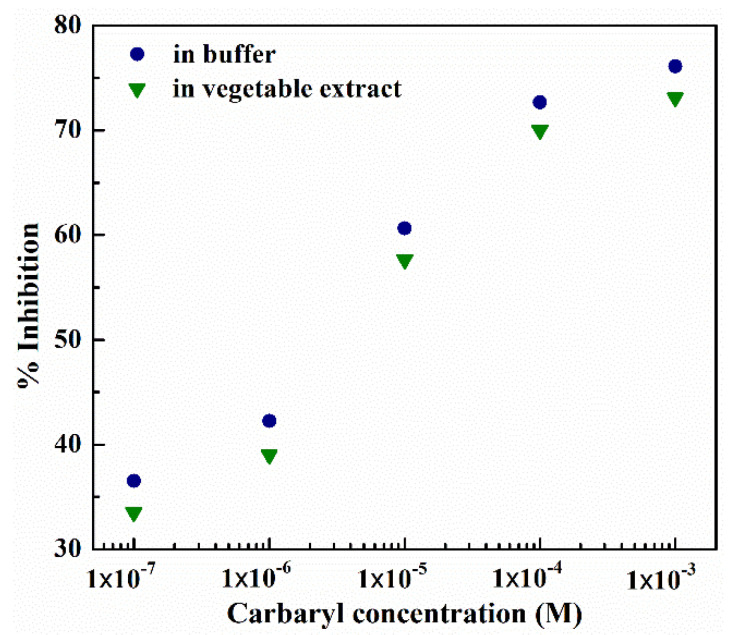
The percentage of enzyme inhibition of PBS- and vegetable extract-diluted carbaryl, given the solution temperature of 25 °C.

To minimize the prediction error, the input of the multiple-input deep learning regression model (i.e., ISFET signal response) needs to be compensated. To calculate the compensated input, we examine the relationship between the percentage of enzyme inhibition of PBS-diluted carbaryl and that of vegetable extract-diluted carbaryl, under variable carbaryl concentrations as shown in Figure 12.

In Figure 12, the percentage of AChE enzyme inhibition of vegetable extract-diluted carbaryl is below that of PBS-diluted carbaryl under all carbaryl concentrations (1 × 10^−7^–1 × 10^−3^ M). Specifically, the percentage of AChE enzyme inhibition of vegetable extract-diluted carbaryl is 2.68–3.24% lower than that of PBS-diluted carbaryl. The lower percentage of enzyme inhibition indicates higher ISFET signal response. As a result, the input (i.e., ISFET signal response) of the multiple-input deep learning regression model has to be compensated by 1.206–1.458 mV, given that 1% enzyme inhibition is equivalent to 0.45 mV (i.e., inverse of Equation (10)).

Table 2 compares the actual carbaryl concentrations in organically grown white cabbage extracts from three vendors of their different supermarkets (vendors 1, 2, and 3) and those predicted by the proposed scheme (i.e., the ISFET sensor with the multiple-input deep learning regression model), given the solution temperatures of 28–32 °C. In vegetable extraction, white cabbage is cut into small shreds of 0.5 × 0.5 cm (W × L) in dimension, extracted by ethanol, and filtered by Whatman No. 1 for vegetable extract.

The experimental solution temperatures of 28–32 °C resemble the ambient temperatures in tropical countries where white cabbage is widely cultivated for domestic consumption and export. The onsite application of the proposed ISFET scheme is carried out in accordance with Figure 7. As shown in Table 2, the discrepancies between the actual and predicted results are negligible, suggesting that the proposed scheme could be deployed onsite (i.e., field test) to efficiently measure the carbaryl concentrations.

## 5. Conclusions

This research proposes an ISFET scheme driven by a multiple-input deep learning regression model with signal compensation to efficiently predict carbaryl concentrations based on the ISFET signal response and the solution temperature. In the experiment, the carbaryl concentrations are varied between 1 × 10^−7^–1 × 10^−3^ M, and the temperatures of solutions were between 20, 25, 30, and 35 °C. The experimental solutions are PBS buffer and AChCl substrate. The MSE, MAE, and R^2^ of the proposed ISFET scheme (i.e., ISFET sensor with the multiple-input deep learning regression model) are 0.007%, 0.016%, and 0.992, indicating very high predictive ability of the proposed ISFET scheme. Further experiments were also carried out whereby the proposed ISFET scheme was deployed in a field test to measure carbaryl concentrations in ethanolic extract of organically grown white cabbage spiked with variable concentrations of the pesticide and results compared with the actual concentrations. The results reveal small discrepancies between the actual and predicted results, indicating the high predictive ability of the proposed ISFET scheme. More importantly, the proposed multiple-input deep learning regression model with signal compensation is applicable to a wide range of solution temperatures in both fractional (e.g., 20.5, 26.7, and 32.6 °C) and non-fractional increments (e.g., 20, 26, and 32 °C) which is facilitated to perform the experiment in field testing with the high predictive ability still be obtained.

In the future development, the data input for the training deep learning regression model should be improved with the field test data (i.e., different environments or different areas) to increase the training datasets and the variability of the data from actual use. Moreover, the proposed ISFET scheme could be utilized to test with the other carbamate pesticides for onsite screening devices.

## Figures and Tables

**Table 1:** Specification of the experimental equipment and sensor.

Equipment/Sensor	Specification
Keysight 34461A Truevolt Digital Multimeter	DCV accuracy 35 ppm Max reading rate 1000 rdgs
Keysight 34461A Truevolt Digital Multimeter	Thermistor 2 wire (10 kΩ)
ISFET sensor	ISFET pH sensor kit (Winsense)

**Table 2:** The actual and predicted carbaryl concentrations using the ISFET sensor with the proposed multiple-input deep learning regression model.

Carbaryl Concentrations in Ethanolic Extract of White Cabbage (M) *	Predicted Carbaryl Concentrations Using the ISFET Sensor with Multiple-Input Deep Learning Regression (M)		
	Vendor 1	Vendor 2	Vendor 3
1 × 10^−7^	0.998 × 10^−7^	0.941 × 10^−7^	0.965 × 10^−7^
1 × 10^−6^	0.966 × 10^−6^	0.977 × 10^−6^	0.958 × 10^−6^
1 × 10^−5^	0.946 × 10^−5^	0.979 × 10^- 5^	0.997 × 10^−5^
1 × 10^−4^	0.955 × 10^−4^	0.957 × 10^−4^	0.968 × 10^−4^
1 × 10^−3^	0.995 × 10^−3^	0.998 × 10^−3^	0.998 × 10^−3^

Note: * The values indicate the actual carbaryl concentrations in ethanolic extracts of organically grown white cabbage from vendors 1, 2, and 3.
